# Pathways from Neighborhood Factors to Adult Criminality: Intervening Roles of Neighborhood Disorder, Juvenile Delinquency, And Violence Exposure

**DOI:** 10.1007/s40865-026-00304-0

**Published:** 2026-04-27

**Authors:** Abigail J Anderson, Christopher C Henrich, Sylvie Mrug

**Affiliations:** University of Alabama at Birmingham, Birmingham, USA

**Keywords:** Neighborhood disadvantage, Neighborhood collective efficacy, Delinquency, Criminal behaviors, Violence exposure, Indirect effects, Moderation

## Abstract

The relationships between neighborhood characteristics, like disadvantage and collective efficacy, and criminal behavior are complex, with few studies examining their longitudinal interplay over the course of adolescence to adulthood. The present study investigated direct, indirect, and interactive effects of neighborhood disadvantage and collective efficacy in adolescence on adult violent and non-violent criminal behaviors (age 16–29), using data from three waves of the Healthy Passages study (*N* = 663). We tested indirect pathways through adolescent delinquency, exposure to neighborhood violence, and perceived neighborhood disorder. Neither neighborhood disadvantage nor collective efficacy directly predicted adult violent or non-violent criminal behaviors. Results indicated that neighborhood disadvantage was associated with higher adolescent exposure to neighborhood violence and greater perceptions of neighborhood disorder, but not with adolescent delinquency. However, an indirect effect emerged, with neighborhood disadvantage related to a lower likelihood of non-violent criminal behavior via greater perceived neighborhood disorder. Adolescent delinquency robustly predicted both violent and non-violent criminal behaviors in adulthood. No significant interactive effects between neighborhood disadvantage and collective efficacy were observed. Findings underscore the complexity of neighborhood influences on crime development and suggest that adolescents’ perceptions of their environments may serve as one potential mechanism linking structural disadvantage to certain long-term behavioral outcomes.

**Supplementary Information:**

The online version contains supplementary material available at 10.1007/s40865-026-00304-0.

The relationships between neighborhood characteristics and adult criminality are complex and not fully understood. A large body of literature has established certain neighborhood factors, such as high crime levels (Assink et al., 2015), community violence (Hoskins et al., 2023), low levels of social cohesion (Farrington et al., 2012), and perceptions of disadvantage (Fowler et al., 2009; Nebbitt & Lombe, 2007; Oh & Connolly, 2022), are risk factors for engagement in delinquent and criminal behaviors in adolescence and adulthood. However, although the concurrent relationship between neighborhood characteristics and criminality has been extensively studied, most studies have only tested cross-sectional relationships (Wenger, 2023). Longitudinal examination is warranted to clarify how these associations unfold over time, allowing for a more nuanced understanding of the complex pathways through which neighborhood factors may shape adult criminality.

Importantly, adult criminal behavior is not a uniform construct and can be broadly distinguished into violent and nonviolent offenses. Violent crimes are directed at individuals and involve the use or threat of force to cause bodily harm, including behaviors such as aggravated assault, fighting, and homicide (Bureau of Justice Statistics, n.d.; Henry et al., 2001). In contrast, nonviolent crimes typically involve property or rule violations, such as theft, drug selling, and burglary. Nonviolent offending is far more common, with most young offenders engaging in some form, whereas only a smaller subset escalate to violent crimes (Cohen & Piquero, 2009). Furthermore, violent and nonviolent offenses may arise from distinct developmental pathways, with neighborhood risk factors exerting differential effects. Examining these outcomes separately may therefore provide greater specificity in understanding how neighborhood disadvantage and social processes shape adult criminality over time.

Prior research has typically considered two types of neighborhood characteristics: neighborhood structure and neighborhood social processes (Leventhal & Brooks-Gunn, 2004). Neighborhood structural characteristics, such as census-tract disadvantage, often include sociodemographic features of a community, such as the median income, employment rate, racial composition, and residential stability. Research has shown that neighborhood-level disadvantage is linked to increased engagement in delinquent and criminal behaviors in adolescence (Chang et al., 2016; Chung & Steinberg, 2006; Fite et al., 2010; Mrug & Windle, 2009), highlighting neighborhood disadvantage as an important risk factor in the development of these behaviors.

In addition to studying neighborhood structural characteristics, research has also examined the impact of neighborhood social ties and processes on the development of delinquency and criminal behaviors (Wickes et al., 2013) guided by the collective efficacy theory (Bandura, 1997). Rooted in theoretical work on self-efficacy, neighborhood collective efficacy refers to the collective action of a neighborhood, with neighborhoods high in collective efficacy having more social ties between residents and adults being more likely to take actions on behalf of the community to reduce delinquent behaviors (Sampson et al., 1997; Simons et al., 2005). Therefore, behavior problems, like delinquency, may result from low levels of social connection in the community and decreased collective efficacy (Simons et al., 2005). Indeed, neighborhood social processes, like collective efficacy, have been found to reduce engagement in criminal behaviors in youth (Meier et al., 2008; Molnar et al., 2008; Sampson et al., 1997).

Much of the literature considering the relationships between neighborhood characteristics and crime has been guided by the Social Disorganization Theory (Shaw & McKay, 1942), which suggests that serious crime flourishes when neighborhood social control breaks down (Kubrin, 2009; Sampson & Groves, 1989; Shaw & McKay, 1942). Extant research on this theory has suggested that neighborhood structural characteristics, like disadvantage and disorganization, weaken the social connections between neighbors, and therefore reduce the ability to build collective efficacy (Kubrin & Wo, 2015). In other words, Social Disorganization Theory posits that neighborhood processes, like collective efficacy, are central to the regulation of crime and disorder (Sampson et al., 1997), with higher levels of collective efficacy related to lower crime rates (Morenoff et al., 2001; Sampson, 2006, 2012; Sampson et al., 1997). Further, higher levels of neighborhood disadvantage, like concentrated poverty and residential mobility, are associated with lower rates of collective efficacy (Sampson et al., 1997). However, although Social Disorganization Theory is often used to explain macro-level crime patterns, its core mechanisms, such as weakened social control and exposure to criminogenic contexts, also suggests that structural disadvantage can influence individual behavior and perceptions by shaping the social and physical environment.

However, some studies have failed to document a direct relationship between these neighborhood factors on delinquency (Bernburg & Thorlindsson, 2007; Maimon & Browning, 2010), indicating that the relationships between neighborhood characteristics, like structural disadvantage and collective efficacy, and criminal behaviors are more complex. As a result, the mechanisms that may contribute to the long-term effects of neighborhoods on criminality are not well understood. It is possible that neighborhood disadvantage and collective efficacy translate into behavioral outcomes not directly, but rather through a series of interrelated mechanisms, including exposure to violence, perceptions of neighborhood disorder, and youth delinquency.

In line with the Social Disorganization Theory (Shaw & McKay, 1942) and a Collective Efficacy Framework (Sampson, 2006), one potential mechanism through which neighborhood structural disadvantage may be related to adult criminal outcomes is through adolescent perceived neighborhood disorder. Understudied compared to other neighborhood characteristics, neighborhood disorder refers to residents’ perceptions of disorganization including crime, drug activity, graffiti, and alcohol use in their living environment, which serve as cues for both physical and social disorder (Coulton et al., 2007; Skogan & Maxfield, 1981; Taylor & Hale, 1986). Previous research has indicated that higher perceived neighborhood disorder is closely related to, and yet distinct from, higher census-tract neighborhood disadvantage (e.g., Mrug & Windle, 2009; Ross et al., 2001). Specifically, neighborhood disadvantage refers to objective structural characteristics of a community, whereas neighborhood disorder reflects residents’ perception of visible physical and social disorganization. Additionally, neighborhood disorder has also been linked to lower neighborhood collective efficacy (Armstrong et al., 2015; Sampson, 2012). With this, higher disadvantage and lower collective efficacy each potentially predispose adolescents to higher perceived physical and social disorder (Ross et al., 2001). However, neighborhood disorder and its role in child outcomes has received relatively less attention, compared with other neighborhood factors, such as neighborhood disadvantage, social cohesion, and social control (e.g., Chung & Steinberg, 2006; Mrug & Windle, 2009). Further, no known studies have considered neighborhood disorder as a mechanism in the relationships between neighborhood disadvantage, collective efficacy, and adult criminal behaviors. Although acting as one piece in a multiple mediator design, previous studies have found that neighborhood structural disadvantage was associated with neighborhood disorder, which was then indirectly related to child externalizing behaviors through parenting (Chung & Steinburg, 2006; Mrug & Windle, 2009; Tolan et al., 2003). Although considering neighborhood disorder as one component within broader mediation pathways, these studies emphasize the importance of considering neighborhood disorder as an intermediary of the relationship between structural disadvantage and adult criminal behaviors.

Another important pathway to consider in the relationship between neighborhood factors and adult criminality is through early juvenile delinquency. Adolescence is a critical period in development where engagement in criminal behaviors becomes more common, with rates typically peaking around 15 years of age (Farrington et al., 2003; Moffitt, 1993; Reyna & Farley, 2006). Juvenile delinquency is often considered one of the best predictors of adult criminality and is often seen as the first step in a criminal career (Basto-Pereira & Farrington, 2022; Farrington et al., 2003; Welsh & Rocque, 2014). Although some studies have not found a direct link (e.g., Maimon & Browning, 2010), a large body of research has supported the relationship between neighborhood characteristics, like disadvantage and collective efficacy, and engagement in delinquency (Haynie et al., 2006; Mrug & Windle, 2009; Van Horn et al., 2007; Wolff et al., 2018). Specifically, heightened disadvantage (Chang et al., 2016; Wolff et al., 2018) and lower collective efficacy (Browning & Erickson, 2009; Molnar et al., 2008) have both been generally linked to higher engagement in delinquent behaviors. Therefore, neighborhood structural disadvantage and neighborhood collective efficacy may each contribute to delinquent behaviors in adolescence that then escalate into adult criminality.

Additionally, neighborhood structural disadvantage and collective efficacy may be related to adult criminality through exposure to violence. Disadvantaged neighborhoods, characterized by poverty and high unemployment rates, are likely to increase exposure of residents to violence due to higher crime rates (Assink et al., 2015; Hoskins et al., 2023). Children, particularly those in areas with higher rates of disadvantage, are exposed to high levels of violence in their communities (Kaynak et al., 2011; McDonald et al., 2011), whereas neighborhoods with higher collective efficacy tend to have lower levels of neighborhood violence (e.g., Ahern et al., 2013; Sampson, 2012; Sampson et al., 1997). Further, several studies have found an association between children’s exposure to violence and engagement in criminal behaviors (Chen et al., 2016; Gorman-Smith et al., 2004).

In summary, neighborhood disadvantage and collective efficacy may be related to adult criminal outcomes indirectly through intervening processes, such as neighborhood disorder, juvenile delinquency, and exposure to violence, which can only be captured using longitudinal data examining these complex relationships over time. In particular, perceived neighborhood disorder, exposure to violence, and delinquency represent theoretically grounded intermediary pathways linking structural context to later offending. Although delinquency, exposure to violence, and perceived neighborhood disorder are conceptually distinct, these experiences are likely interrelated during adolescence and may co-occur, especially within disadvantaged environments. Examining these pathways simultaneously allows for the assessment of their unique contributions while accounting for their overlap, providing insight into how neighborhood factors may be related to adult criminality through developmentally proximal adolescent experiences.

Beyond its direct and indirect associations with adult criminality, collective efficacy may also function as a moderator. However, the potential protective role of collective efficacy in buffering the effects of neighborhood disadvantage on violence exposure, delinquency, perceived neighborhood disorder, and later adult criminal outcomes remains largely understudied. Using the Social Disorganization framework, collective efficacy may not only shape crime rates directly but may also moderate broader structural disadvantage. Previous studies have theorized the presence of collective efficacy in communities may attenuate, or buffer, the negative effect of neighborhood disadvantage on criminal behaviors over time (Aisenberg & Herrenkohl, 2008), with one study indicating that collective efficacy was a buffer in the relationship between violent victimization and substance use in adolescents (Fagan et al., 2014). In other words, collective efficacy may serve as a community-level buffer where strong interpersonal networks and trust can offset the adverse consequences associated with structural disadvantage. Further, this notion aligns with broader stress-buffering hypothesis (Cohen & Wills, 1985), which proposes that social resources can mitigate the adverse effects of environmental stressors. In disadvantaged neighborhoods, high collective efficacy may help maintain social control, reinforce prosocial norms, and provide youth with role models or supports that reduce the likelihood of engaging in delinquent acts. In line with this framework, one study found that the effects of economic disadvantage on antisocial traits were attenuated in neighborhoods high in collective efficacy (Odgers et al., 2009). Despite the potential protective nature of collective efficacy, its moderating role in buffering the effects of neighborhood structural disadvantage on perceived neighborhood disorder, violence exposure, delinquency, and adult criminal offending remains understudied.

## The Current Study

**Fig. 1:**
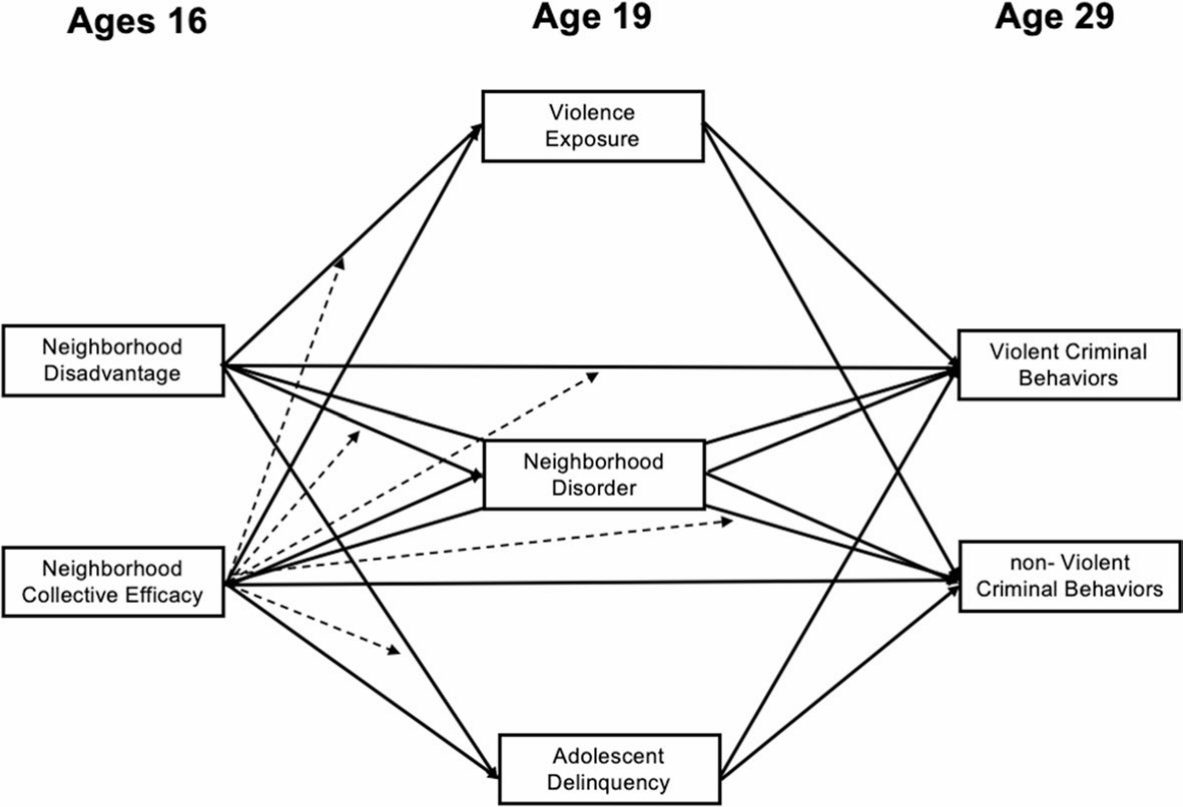
Hypothesized path model. *Note*: Solid arrows represent main effects; dashed arrows represent paths tested for moderation. All interactions were estimated within the structural model, but interaction terms are shown conceptually for clarity

A large body of research has considered the concurrent relationship between neighborhood disadvantage and criminality. However, longitudinal investigation of the mechanisms that may contribute to that relationship is warranted (Airaksinen et al., 2021; Kubrin & Weitzer, 2003; Wenger, 2023), as longitudinal analyses allow for the testing of complex, developmental pathways that prior studies using concurrent data could not test. To address this gap, we tested (Fig. 1) indirect effects of neighborhood characteristics, such as disadvantage and collective efficacy, on subsequent criminal behaviors through adolescent delinquency, perceived social disorder, and exposure to violence. In line with findings from previous studies, neighborhood disadvantage was expected to be related to subsequent adolescent delinquency (Chang et al., 2016; Kim et al., 2019; Wolff et al., 2018) and adult criminality. Given that higher rates of violence are concentrated in disadvantaged neighborhoods (De Coster et al., 2006; Friedson & Sharkey, 2015), neighborhood disadvantage was also expected to be related to adolescent’s exposure to violence. Further, we expected that higher census-tract disadvantage would be related to higher perceptions of neighborhood disorder. We also hypothesized that adolescent delinquency, which is considered one of the best predictors of criminal behaviors in adulthood (Bergman & Andershed, 2009; Welsh & Rocque, 2014), and adolescent violence exposure would be related to subsequent engagement in criminal behaviors. Although delinquency, exposure to violence, and perceived neighborhood disorder are conceptually distinct, these experiences are likely interrelated in adolescence. Therefore, delinquency, perceived neighborhood disorder, and exposure to violence were each expected to partially explain the relationship between neighborhood characteristics and adult criminality. Further, given its potential protective nature, we also expected that neighborhood collective efficacy would buffer the adverse effects of neighborhood disadvantage on adolescent delinquency, perceived neighborhood disorder, adolescent exposure to violence, and adult engagement in criminal behaviors.

## Method

### Participants and Procedures

Participants were drawn from the Birmingham site of the Healthy Passages Study, which is a longitudinal study of adolescent health and development. At baseline, 1597 fifth-grade students (Wave 1; *Mage* = 11.2 years, *SD* = 0.53: 50% Female, 50% male, 56% Black or African American; 44% White/Other) and their primary caregivers were recruited using a two-stage probability sampling design (Coker et al., 2009; Windle et al., 2004). Participants were interviewed at five time points from early adolescence into adulthood, with follow-ups occurring at age 13 (Wave 2, *N* = 1535; *Mage* = 13.1 years, *SD* = 0.55), age 16 (Wave 3, *N* = 1392; *Mage* = 16.2 years, *SD* = 0.54), age 19 (Wave 4, *N* = 1273; *Mage* = 19.7 years, *SD* = 1.42), and age 29 (Wave 5, *N* = 817; *Mage* = 28.8 years, *SD* = 1.05) for which data collection is ongoing. The analytic sample of participants included 663 adults (44% Male, 56% Female, 64% African American, 36% White/Other) who completed interviews at ages 16, 19, and 29.

At child age 16, each child and primary caregiver were interviewed separately in private spaces. At age 19 and 29, only the adolescent or young adult was interviewed. For participants younger than 18 years, a primary caregiver signed informed consent, and the child provided a voluntary agreement to participate and signed an assent. Participants over 18 years old signed informed consent prior to being interviewed. Participant interviews were conducted in private spaces by a trained staff with computer-assisted personal interviews (CAPI; Herek & Capitanio, 1996), with sensitive questions asked via an audio-computer-assisted self-interview (CASI). All participants received monetary compensation in the form of a reloadable prepaid debit card for their time. To assess neighborhood characteristics, participants’ addresses from age 16 were geocoded and linked to the 2010 US Census Bureau data.

To account for attrition earlier in the study, we examined missingness at age 29 using logistic regression in SPSS version 29 to test whether demographic characteristics and main study variables at age 16 and 19 differed between participants retained versus not retained. Results indicated that participants not retained at age 29 did not differ on neighborhood disadvantage, neighborhood collective efficacy, participant race, parent marital status, primary caregiver education, nor household income. However, results of the logistic regression revealed that males, *OR* = 0.59, CI95%[0.44; 0.79], were less likely to return by age 29 than females.

## Measures

### Neighborhood Disadvantage

At age 16, participant addresses were geocoded to obtain census tract information from the 2010 decennial U.S. census data and were linked to their corresponding neighborhood block group. Neighborhood Disadvantage was measured using the Area Deprivation Index (ADI) (Kind & Buckingham, 2018; University of Wisconsin School of Medicine and Public Health, 2015). The ADI uses 17 socioeconomic measures from US Census data to rank neighborhoods, with indicators weighted using factor score coefficients that give poverty, income, and education variables the largest relative weights. The Census variables are multiplied by their factor weights and then a sum is provided for each geographic unit (Kind & Buckingham, 2018). It was developed and validated in the United States to provide a composite index of disadvantage by neighborhood, with scores ranging from 1 to 10 at national and state levels, with higher scores indicating more disadvantage. As data was collected within a single state, the ADI state decile rankings were used. In the current sample, 260 neighborhood block clusters were utilized, with an average of 2.55 participants per cluster.

### Neighborhood Collective Efficacy

At age 16, parents reported on neighborhood collective efficacy with two scales from the Project on Human Development in Chicago Neighborhoods Community Survey Questionnaire (Earls, 1999): Informal Social Control (5 items) and Neighborhood Social Exchange (5 Items). The informal social control scale was measured using a 5-point scale ranging from “Very Unlikely” to “Very Likely” to assess the likelihood that neighborhood residents would intervene if neighborhood children were skipping school, defacing a local building with graffiti, showing disrespect to adults, fighting in front of one’s house, or if a local fire station was threatened with closure. The Informal Social Control scale demonstrated good internal consistency (α = 0.85). The Neighborhood Social Exchange scale asked parents to rate the frequency in which neighbors did favors for each other, watched each other’s home, asked for advice from neighbors, had parties or get-togethers, or visited with neighbors. Items were rated on a 4-point scale ranging from “Never” to “Often”, with the measure reflecting good internal consistency (α = 0.83). Items on each scale were summed, with a moderate correlation between the two scales (*r*=.48, *p*<.001). The two scales were averaged as an estimate of collective efficacy, in line with prior research conceptualizing collective efficacy as a combination of social cohesion and informal social control. Higher scores indicate higher levels of collective efficacy.

### Neighborhood Violence Exposure

At age 19, adolescents were asked how frequently they had witnessed or were victimized by violence in their neighborhood within the past twelve months, including (1) threats of violence, (2) physical violence, and (3) threats of or physical violence involving a weapon. Participants rated items on a four-point frequency scale ranging from “Never” to “Many Times”/ Responses were dichotomized such that “Never” was coded as no exposure (0), and any reported exposure (“Once,” “A Few Times,” or “Many Times”) was coded as exposure (1). The items were then summed to obtain a total Neighborhood Violence exposure score, with the current sample demonstrating adequate reliability (α = 0.70). However, because this composite combines forms of violence exposure that are conceptually separate and unlikely to co-occur, for example, witnessing threats of violence in the neighborhood does not imply direct victimization involving a weapon, indices of internal consistency (e.g., Cronbach’s alpha) are not suitable for assessing its reliability (Kalkbrenner, 2023).

### Perceived Neighborhood Disorder

At age 19, the adolescents reported on neighborhood disorder using 11 items from the Neighborhood/Block Conditions scale (Perkins et al., 1990). Items probed the degree of social (e.g., drug dealing, organized gangs) and physical (e.g., property damage, cleanliness) disorder in their neighborhood. Each item was rated on a 4-point scale from “Strongly Disagree” to “Strongly Agree”. Responses to all items were summed, with higher scores indicating higher levels of perceived neighborhood disorder. In the current sample, perceived neighborhood disorder evidenced good reliability (α = 0.91).

### Adolescent Delinquency

At age 19, adolescents reported the frequency they engaged in different delinquent acts in the last 12 months. The 13 items used were adapted from Elliott et al. (1985) and probed how often participants engaged in behaviors such as fighting, stealing or shoplifting, breaking into a building or vehicle, or selling drugs. Items were dichotomized to reflect if the adolescent engaged in that specific behavior (Yes = 1, No = 0). Items were then summed to create a variety score, with higher scores indicating engagement in a broader range of delinquent behaviors. In the current sample, delinquency demonstrated good reliability (α = 0.80).

### Criminal Behaviors

At age 29 participants reported the frequency they engaged in criminal behaviors with the same 13 items used at age 19, which were adapted from Elliott et al. (1985). Items were divided into violent (6-items; α = 0.77) and non-violent (7-items; α = 0.77) categories. Violent criminal behaviors included items such as fighting, hitting, cutting or stabbing someone, whereas non-violent criminal behaviors included items such as stealing, selling drugs, and breaking into a building or vehicle. Due to low endorsement of individual items at age 29, all responses were subsequently dichotomized to indicate whether participants had engaged in any violent criminal behaviors (Yes = 1, No = 0) or nonviolent criminal behaviors (Yes = 1, No = 0).

### Covariates

Child sex (Male = 1, Female = 0) and race (Black = 1, White/Other = 0) were included as covariates. In order to account for earlier levels, neighborhood violence exposure was included as a covariate. At age 16, using the same measure as used for age 19, adolescents reported how often they had witnessed or experienced neighborhood violence in the past 12 months, including threats of violence, physical violence, and violence involving a weapon. Items were rated on a four-point scale (“Never” to “Many Times”) and dichotomized such that any exposure was coded as 1 and no exposure as 0. Items were summed to create a total neighborhood violence exposure score (α = 0.68). Further, early problem behaviors were assessed at age 16, with items from the Diagnostic Interview Scale for Children (DISC) predictive scales (Lucas et al., 2001). Adolescents reported whether or not they engaged in 15 behaviors (Yes = 1, No = 0) in the past 12 months with items from the Conduct and Oppositional Defiant Disorder subscales. Example items include: “gotten even with people”, “done mean things to people on purpose”, and “been physically cruel to an animal”. Items were then summed, with higher scores indicating higher reported delinquency. In the current sample, delinquency at age 16 had acceptable reliability (α = 0.79).

Further, analyses controlled for parent marital status (1 = Married, 0 = Not Married), primary caregiver education (1 = Above High school, 0 = Below high school), and primary caregiver age at child age 16. Lastly, we included family income at age 16 as a covariate, which was measured using an ordinal scale with 20 categories (1=<$5000 to 20 = > 250,000).

### Analytic Strategy

Preliminary analyses included examining descriptive statistics, including means and standard deviations, and bivariate correlations. As shown in Fig. 1, our primary analysis tested an integrated path model utilizing three waves of data from the Healthy Passages Study. This model simultaneously examined the direct effects of neighborhood disadvantage and collective efficacy in adolescence on adult violent and nonviolent criminal behaviors, as well as the indirect effects through adolescent delinquency, neighborhood violence exposure, and perceived neighborhood disorder. Main analyses were conducted in Mplus v8.8 with Full Information Maximum Likelihood (FIML) Estimation with robust standard errors to handle missing data and non-normally distributed variables while minimizing bias and preserving sample size (Graham et al., 2013; Wothke, 2000), as well as Montecarlo Integration. To account for the nonindependence of participants residing within the same neighborhoods, all models were estimated with adjustment for standard errors by clustering within neighborhood block groups using TYPE=COMPLEX.

To assess the relationships over time, we simultaneously conducted linear regressions to appropriately model the continuous mediators and binary logistic regressions to model the dichotomous nature of engagement in violent and non-violent criminal behaviors. We also tested the interactive effects of neighborhood disadvantage and neighborhood collective efficacy on later neighborhood disorder, violence exposure, delinquency, as well the likelihood of violent and non-violent adult criminal behaviors. All paths were adjusted for the covariates of early delinquency, early violence exposure, child sex, child race, parent marital status, primary caregiver age, primary caregiver education, and family income.

Both census-tract neighborhood disadvantage and neighborhood collective efficacy were mean centered prior to creating the interaction term. Any identified interaction effects were probed with simple slopes one standard deviation above and below the mean of the moderator, and for binary outcomes, marginal effects were calculated to interpret how the predicted probability of the outcome changes across levels of the interacting variables. Indirect effects were tested with bias-corrected bootstrapping with 1000 bootstrap samples (Muthén & Muthén, 2017). Model fit was assessed with comparative fit index (CFI) and root mean square error of approximation (RMSEA), with CFIs greater than 0.95 and RMSEA less than 0.06 indicating good model fit (Hu & Bentler, 1999).

## Results

### Descriptive and Bivariate Associations

Descriptive statistics are reported in Table 1 and bivariate correlations among study variables are reported in Table 2. On average, participants resided in moderately disadvantaged neighborhoods, with a mean state-ranked ADI score of 5.70. At age 19, participants engaged in an average of 1.62 delinquent behaviors, indicating a generally low level of delinquency in this sample. Approximately 15% of participants reported engaging in violent criminal behaviors at age 29, with approximately 30% of participants reported engaging in non-violent criminal behaviors. Neighborhood disadvantage at age 16 was negatively correlated with neighborhood collective efficacy. However, neighborhood disadvantage at age 16 was positively related to perceived neighborhood disorder and neighborhood violence exposure at 19. Conversely, neighborhood collective efficacy at age 16 was negatively correlated with violence exposure and perceived neighborhood disorder at age 19. Violence exposure, delinquency, and neighborhood disorder were all positively correlated at age 19. Delinquency and neighborhood violence exposure at age 19 were positively related with both violent and non-violent criminal behaviors at age 29. However, perceived neighborhood disorder was only correlated with engagement in violent criminal behaviors, not non-violent criminal behaviors.

**Table 1:** Descriptive statistics

Variable	%	Mean	SD	Min	Max
*Outcome Variable*					
Violent Criminal Behavior (Age 29)	14.6			0	1.00
Non-Violent Criminal Behavior (Age 29)	29.7			0	1.00
*Explanatory Variables*					
Neighborhood Disadvantage (Age 16)		5.70	3.52	0	10.00
Neighborhood Collective Efficacy (Age 16)		16.28	3.61	0	24.00
Delinquency (Age 19)		1.62	2.02	0	13.00
Violence Exposure (Age 19)		0.53	1.07	0	6.00
Perceived Neighborhood Disorder (Age 19)		19.41	6.71	11	44
*Covariates*					
Delinquency (Age 16)		3.01	2.69	0	15
Violence Exposure (Age 16)		0.50	1.03	0	6
Child Sex (Male)	44.2			0	1.00
Child Race (Black)	64.4			0	1.00
Family Income		8.12	5.40	0	1.00
Parent Marital Status (Married; Age 16)	51.9			0	1.00
Primary Caregiver Education (Above High School; Age 16)	68.0			0	1.00
Primary Caregiver Age		42.55	7.06	27.00	70.00

**Table 2:** Bivariate Correlations among key variables

1. *N* Disadvantage (Age 16)	1.	2.	3.	4.	5.	6.	7.
2. N Collective Efficacy (Age 16)	− .38***						
3. Delinquency (Age 19)	.08*	− .04					
4. Violence Exposure (Age 19)	.26***	− .15***	.43***				
5. Neighborhood Disorder (Age 19)	.46***	− .27***	.30***	.43***			
6. Violent Criminal Behaviors (Age 29)	.14***	− .12**	.23***	.22***	.19***		
7. Non-Violent Criminal Behaviors (Age 29)	.11**	− .09*	.20***	.18***	.05	.39***	

*p<.05, **p<.01, ***p<.001

### Main Analysis

Unstandardized results are presented in Table 3. The model explained 17% of variance in adolescent delinquency, 21% of variance in neighborhood violence exposure, and 31% in perceived neighborhood disorder. Neither neighborhood disadvantage nor neighborhood collective efficacy measured at age 16 was directly related to the likelihood of engaging in violent or non-violent criminal behaviors at age 29. However, higher neighborhood disadvantage at age 16 was related to higher subsequent reports of neighborhood violence exposure and higher neighborhood disorder at age 19 but was not related to subsequent adolescent delinquency. Neighborhood collective efficacy at age 16 was not related to later violence exposure, delinquency, nor was it related to perceived neighborhood disorder. However, a standard deviation increase in neighborhood violence exposure at age 19 was associated with a 14% increase in the odds of engaging in non-violent criminal behaviors at age 29. Delinquency at age 19 was also related to the likelihood of both violent and non-violent criminal behaviors at age 29, with a standard deviation increase in delinquency associated with a 22% increase in the odds of violent criminal behaviors and a 28% increase in the odds of engaging in non-violent criminal behaviors. A standard deviation increase in perceived neighborhood disorder was associated with a 14% decrease in the odds of non-violent criminal behaviors. No statistically significant interactions were observed.

**Table 3:** Unstandardized regression coefficients

	Violence Exposure (Age 19)	Delinquency (Age 19)	Neighborhood Disorder (Age 19)	Violent Criminal Behaviors (Age 29)			Non-Violent Criminal Behaviors (Age 29)		
	b(SE)	b(SE)	b(SE)	b(SE)	OR	95% CI	b(SE)	OR	95% CI
*Covariate Effects*									
Violence Exposure (Age 16)	0.25 (.06)***	0.48 (.16)**	1.24 (.02)***						
Delinquency (Age 16)	0.06 (.01)**	0.20 (.04)***	0.24 (.01)**						
Child Sex (Male)	0.38 (.08)***	0.62 (.16)***	1.67 (.04)***	0.14 (.28)	1.15	[0.65, 1.92]	-0.28 (.19)	0.76	[0.51, 1.04]
Child Race (Black)	0.09 (.10)	0.00 (.24)	1.52 (.06)*	0.75 (.50)	2.11	[0.88, 5.96]	0.45 (.30)	1.57	[0.85, 2.54]
Family Income (Age 16)	-0.01 (.01)	0.02 (.02)	-0.09 (.01)	-0.02 (.04)	0.97	[0.91, 1.05]	-0.01 (.03)	.992	[0.94, 1.04]
Parent Marital Status (Age 13)	0.07 (.09)	-0.26 (.20)	-0.29 (.05)	-0.28 (.29)	0.75	[0.45, 1.37]	0.13 (.23)	1.14	[0.70, 1.65]
PC Education (Above High School)	-0.16 (.11)	-0.02 (.21)	-0.67 (.04)	0.22 (.28)	1.24	[0.72, 2.27]	0.13 (.24)	1.13	[0.71, 1.04]
Parent Age	-0.01 (.01)	-0.02 (.02)	0.04 (.00)	-0.03 (.02)	0.97	[0.93, 1.00]	0.02 (.01)	1.02	[0.99, 1.04]
*Hypothesized Effects*									
Disadvantage (Age 16)	0.03 (.02)*	0.03 (.04)	0.46 (.01)***	-0.03 (.06)	0.97	[0.88, 1.10]	0.03 (.04)	1.03	[0.95, 1.12]
Collective Efficacy (Age 16)	0.00 (.01)	0.02 (.03)	-0.12 (.01)	-0.03 (.03)	0.97	[0.90, 1.04]	-0.04 (.03)	0.96	[0.90, 1.02]
Disadvantage x Collective Efficacy	-0.00 (.00)	-0.02 (.01)	-0.01 (.00)	0.02 (.01)	1.02	[1.00, 1.04]	0.01 (.01)	1.01	[0.10,1.03]
Violence Exposure (Age 19)				0.14 (.14)	1.16	[0.90, 1.55]	0.23 (.11)*	1.26	[1.03, 1.50]
Delinquency (Age 19)				0.18 (.06)**	1.20	[1.08, 1.34]	0.22 (.06)***	1.25	[1.09, 1.36]
Neighborhood Disorder (Age 19)				0.01 (.27)	1.01	[0.97, 1.06]	-0.04(.25)*	0.96	[0.92, 1.00]

*p<.05, **p<.01, ***p<.001

### Indirect Effects

All indirect effects are reported in Supplemental Materials. Only one significant indirect effect emerged in this model. Higher neighborhood disadvantage at age 16 was indirectly related to lower likelihood of engaging in non-violent criminal behaviors at age 29 via higher perceived neighborhood disorder at age 19, β=-0.04, SE=0.02, *p*=.032 CI95%[-0.09, − 0.01]. However, no other significant indirect effects from either neighborhood disadvantage or collective efficacy to the likelihood of criminal behaviors through adolescent delinquency, neighborhood violence exposure, or perceived neighborhood disorder were found as the required associated between the study variables were not observed.

### Covariate Effects

Higher early delinquency at age 16 was related to higher subsequent engagement in delinquent behaviors, higher neighborhood violence exposure, as well as higher perceived neighborhood disorder at age 19. Higher neighborhood violence exposure at age 16 was related to subsequent reports of neighborhood violence exposure, higher delinquency at age 19, and higher perceived neighborhood disorder. Further, males reported higher violence exposure, higher engagement in delinquency, and higher perceived neighborhood disorder. Child sex was not significantly related to the likelihood of engaging in violent nor non-violent criminal behaviors in adulthood. However, child race was only significantly related to perceived neighborhood disorder, with black participants reporting more disorder. Family income, parent age, marital status, and education were not significantly related to any study outcomes.

## Discussion

We used a three-wave longitudinal design to examine the direct and interactive pathways from neighborhood disadvantage and collective efficacy in adolescence to the likelihood of adult criminal behaviors, considering indirect effects through adolescent delinquency, perceived neighborhood disorder, and neighborhood violence exposure. Guided by the Social Disorganization Theory (Shaw & McKay, 1942) and Collective Efficacy Theory (Sampson et al., 1997), this study sought to examine how neighborhood structural characteristics and social processes were related to criminal behaviors over the course of adolescence to adulthood. Although most associations were non-significant, the pattern of findings raises important questions about how both neighborhood perception of disorder and structural disadvantage operate across developmental stages. Rather than clarifying long-term pathways, the results suggest that the processes linking adolescent neighborhood context to adult offending may be more contingent and developmentally dynamic than previously assumed.

### Role of Neighborhood Disadvantage

The results highlight the role of neighborhood disadvantage in shaping adolescents’ experiences and perceptions of their environment. Neighborhood disadvantage was associated with subsequent exposure to neighborhood violence exposure and greater perceptions of perceived neighborhood disorder. However, contrary to expectations, neighborhood disadvantage was not related to adolescent self-reported delinquency, in line with other studies that have failed to document a direct effect of neighborhood disadvantage on delinquency (e.g., Maimon & Browning, 2010). The lack of findings in this study may suggest that while disadvantage shapes contextual exposures and perceptions, its translation into behavioral risk may depend on more proximal individual or family-level processes, such as through parenting style (e.g., Mrug & Windle, 2009) or peer behaviors (e.g., Goetz et al., 2025). Additionally, this pattern suggests that life-course criminological and neighborhood theories may require refinement when applied to relatively low-risk samples or extended developmental intervals. It is possible that neighborhood effects are strongest during adolescence, when peer influence and routine activities are most neighborhood-bound, and attenuate as individuals transition into adulthood and experience greater mobility and autonomy.

Further, a significant indirect effect emerged, indicating that higher neighborhood disadvantage was indirectly related to a lower likelihood of non-violent criminal behavior via higher perceived neighborhood disorder. The direction of this indirect pathway was opposite to what was hypothesized and therefore warrants cautious interpretation. The positive association between neighborhood structural disadvantage and perceived disorder is consistent with prior research (Mrug & Windle, 2009; Ross et al., 2001). Notably, however, higher neighborhood disorder was associated with a lower likelihood of non-violent offending in adulthood, in contrast to expectations. Although somewhat counterintuitive, this pattern could suggest that when adolescents perceive more disorder in their communities, they may see less opportunities for non-violent criminal behaviors, such as theft or property damage. Further, in less advantaged neighborhoods, there may be more levels of community control, such as policing or surveillance (Kirk, 2008), which may inadvertently create a sense of increased risk associated with criminal behaviors in both adolescents and adults (Kirk & Matsuda, 2011), thus reducing their engagement in criminal behaviors. Another explanation for the opposite to hypothesized direction may be the substantial time gap between the assessment of neighborhood disorder in adolescence and adult criminal behavior, nearly a decade later. Adolescents who perceived high levels of disorder may not have remained in those same environments into adulthood, and residential mobility, changing neighborhood conditions, or transitions into new social roles (e.g., employment, independent living) may have altered their exposure to risk. It is also possible that early awareness of disorder heightened vigilance or motivated efforts to disengage from risky contexts over time, which was not able to be measured. Thus, the extended developmental interval between measurement points may have attenuated or reshaped the association, making the long-term direction of neighborhood effects less clear. As such, strong conclusions regarding long-term neighborhood pathways remain premature.

From a developmental perspective, this study underscores the importance of adolescence as a formative period for exposure to neighborhood risks and emergence of criminality that may be maintained through adolescence and into adulthood. In line with expectations and previous literature (Farrington et al., 2003; Welsh & Rocque, 2014), adolescent delinquency was a robust predictor for both violent and non-violent criminal behaviors in adulthood, underscoring the developmental continuity of problem behaviors across the lifespan. However, neighborhood violence exposure only predicted greater odds of engaging in non-violent criminal behaviors, suggesting that chronic exposure to neighborhood violence may not normalize overt aggression over time.

### Role of Collective Efficacy

Despite theoretical support for collective efficacy as a protective social factor (Sampson, 2006; Wickes et al., 2013), the current study did not find any significant direct or interactive effects of collective efficacy on adolescent or adult criminal outcomes. The absence of significant main or interactive effects may suggest that collective efficacy may only be protective in certain neighborhood contexts, with collective efficacy potentially exerting a stronger influence in high-risk or more highly disadvantaged neighborhoods than those represented in relatively moderate-risk sample. Our null effects for collective efficacy suggest that its influence over time may be more complex than initially presumed, aligning with arguments that collective efficacy is an ecological theory of crime intended to explain community-level, rather than individual-level, variation in crime (Sampson, 2012; Sampson et al., 2005). Additionally, the current study utilized two scales, capturing social resources between neighbors and informal social control, to measure collective efficacy rather than a singular measure, which may account for the absence of findings. Notably, collective efficacy was measured with parent report, which may differ from adolescent’s perception of their neighborhood’s collective efficacy which may be more impactful on their behaviors. Additionally, although not a hypothesized path, the absence of sex differences in violent and non-violent offending at age 29 is notable, as it diverges from robust evidence documenting higher rates of offending among men (e.g., Mallicoat, 2022; Steffensmeier & Allan, 1996). In the current sample, males reported higher engagement in delinquency during adolescence than females, in line with expectations. Several explanations may account for this developmental pattern. First, the relatively low base rates of adult offending in this community-based sample may have reduced variability, limiting the detection of sex differences. Second, in line with our lack of findings for nonviolent behaviors, sex gaps in offending tend to be narrower in adulthood for nonviolent crimes, such as property and drug related offenses (Mallicoat, 2022). As such, the absence of sex differences in adult criminal offending should be interpreted cautiously, as it may reflect developmental, contextual, or measurement factors rather than true convergence.

### Strengths, Limitations, and Future Directions

The present study included several strengths, such as the examination of direct, indirect, and interactive effects over the course of 13 years. A key strength was the inclusion of a three-wave design which allowed for the temporal separation between our predictors, mediators, as well as our outcomes. However, our findings should be considered within the context of our limitations. One major limitation revolves around the measure of our outcome variables, engagement in violent and non-violent criminal behaviors, which was limited to whether or not the individual had participated in any criminal behaviors. Future research should consider a broader range of criminality to capture more nuanced patterns, such as measuring criminal attitudes (Mills et al., 2002) and perceived arrest likelihood (Thomas & Baumer, 2025). Additionally, although the incorporation of three timepoints was a strength, the nearly 10-year time gap between the age 19 and 29 measurements may have attenuated associations over time, with the processes between the included variables potentially unfolding over shorter timescales. Future studies should incorporate more frequent measurements of the variables to assess how neighborhood perceptions measured during adolescence translate into criminal behaviors over time. Moreover, given the use of a low risk sample, as seen with the low levels of delinquency, criminal behaviors, and violence exposure, the ability to detect meaningful developmental pathways may have been limited in the current study. Further, given limitations in the data set, we were unable to control for earlier levels of perceived neighborhood disorder, as recommended for longitudinal mediation (Cole & Maxwell, 2003; Selig & Preacher, 2009; Caemmerer et al., 2024), which we did for delinquency and neighborhood violence exposure. Future studies should include repeated measures to isolate the effects of neighborhood disadvantage and collective efficacy on change in subsequent perceived disorder. Further, the study included a relatively low-risk sample, and the results may not generalize to higher-risk or justice involved populations. Future research should consider how neighborhood processes may operate differently in populations exposed to higher levels of disadvantage, violence, as well as those with greater criminal involvement. Additionally, future studies should continue to investigate the mechanisms involved in the relationships between neighborhood disadvantage, neighborhood collective efficacy, and future criminal behaviors, but broaden the scope to include other potential mediating factors such as parenting (e.g., Mrug & Windle, 2009) or peer deviancy (e.g., Goetz et al., 2025).

### Implications

Our results contribute to the growing understanding of how neighborhood-level risk processes operate across development, emphasizing the importance of adolescence as a sensitive period in which neighborhood exposures and perceptions begin to shape behavioral trajectories (e.g., Levanthal & Brooks-Gunn, 2000). In line with recommendations from previous research (Sharkey, 2018; Wickes et al., 2013), the present findings raise new questions and highlight the need for continued longitudinal research that examines the dynamic interplay among neighborhood disadvantage, perceptions of neighborhood conditions, and social control mechanisms in shaping behavioral outcomes across development, as cross-sectional studies may fail to capture the complex processes involved.

## Conclusions

In conclusion, this study extends prior work on neighborhood factors and criminality by testing a longitudinal model that incorporates both structural indicators of neighborhood disadvantage and perceptual assessments of collective efficacy and neighborhood disorder, capturing multiple pathways from adolescence into early adulthood. Although neighborhood disadvantage was not directly linked to adult criminal behaviors, its associations with adolescents’ exposure to violence and perceptions of neighborhood disorder highlight how neighborhood contexts may shape developmental risk factors over time. Moreover, the absence of significant effects for collective efficacy and its interaction with neighborhood disadvantage suggests that its protective influence may depend on measurement approach, developmental timing, or broader neighborhood context. The findings underscore the importance of considering both objective and subjective dimensions of neighborhood environments when examining pathways to criminality, as adolescents’ interpretations of their surroundings may play a pivotal role in shaping later outcomes. Future research should continue to investigate these complex, multilevel mechanisms to inform community-based prevention and policy efforts aimed at reducing long-term criminal behavior.

## Supplementary Information

Below is the link to the electronic supplementary material.

Supplementary Material 1

## Data Availability

The datasets analyzed during the current study are not publicly available but are available from the corresponding author on reasonable request.
