# Supplementary material for: Pathways from Neighborhood Factors to Adult Criminality: Intervening Roles of Neighborhood Disorder, Juvenile Delinquency, And Violence Exposure
Source: J Dev Life Course Criminol. 2026 Apr 27;12(1):15. doi: 10.1007/s40865-026-00304-0 (PMC13121307; doi:10.1007/s40865-026-00304-0)
Supplement: Supplementary file 1 — Supplementary Material 1 [file 40865_2026_304_MOESM1_ESM.docx]

Standardized Indirect Effects from Neighborhood Disadvantage and Collective Efficacy to Violent and non-Violent criminal behaviors

| Specific Indirect | β | SE | p | CI95% |
| --- | --- | --- | --- | --- |
| Neighborhood Disadvantage🡪 Delinquency 🡪 Violent Criminal Behavior | .01 | .02 | .527 | [-.02, .04] |
| Neighborhood Disadvantage🡪 Violence Exposure🡪 Violent Criminal Behavior | .01 | .01 | .366 | [-.00, .03] |
| Neighborhood Disadvantage🡪 Neighborhood Disorder🡪 Violent Criminal Behavior | .01 | .02 | .541 | [-.02, .05] |
| Neighborhood Disadvantage🡪 Delinquency 🡪 Non-Violent Criminal Behavior | .01 | .02 | .511 | [-.02, .05] |
| Neighborhood Disadvantage🡪 Violence Exposure 🡪 Non-Violent Criminal Behavior | .01 | .01 | .147 | [.00, .04] |
| Neighborhood Disadvantage🡪 Neighborhood Disorder 🡪 Non-Violent Criminal Behavior | -.04 | .02 | .037 | [-.08, -.01] |
| Neighborhood Collective Efficacy🡪 Delinquency 🡪 Violent Criminal Behavior | .01 | .01 | .571 | [-.01,.03] |
| Neighborhood Collective Efficacy🡪 Violence Exposure🡪 Violent Criminal Behavior | .00 | .00 | .995 | [-.01, .01] |
| Neighborhood Collective Efficacy🡪 Neighborhood Disorder🡪 Violent Criminal Behavior | -.00 | .01 | .586 | [-.02,.01] |
| Neighborhood Collective Efficacy🡪 Delinquency 🡪 Non-Violent Criminal Behavior | .01 | .02 | .562 | [-.02, .04] |
| Neighborhood Collective Efficacy🡪 Violence Exposure 🡪 Non-Violent Criminal Behavior | .00 | .01 | .994 | [-.01, .01] |
| Neighborhood Collective Efficacy🡪 Neighborhood Disorder 🡪 Non-Violent Criminal Behavior | .01 | .02 | .200 | [.00, .03] |
